# Editorial

**Published:** 1864-09

**Authors:** 


C. S. Medical & Surgical Journal.

RICHMOND, SEPTEMBER, 1864.

*AYRES & WADE......PUBLISHERS AND PROPRIETORS.*

### EDITORIAL.

*On the Treatment of Gun-Shot Wounds by "Hermetically Sealing."*

In one of the earlier numbers of this Journal, (Vol. I., No. 4,) the attention of our readers was directed to a proposal of Assistant-Surgeon Howard, of the United States Army, first published in the "New York Medical Times," to treat gun-shot wounds of the chest by hermetically sealing the orifices of entrance and exit, and by thus preventing the entry of air into the cavity of the thorax, hemorrhage would be controlled, dyspnœa relieved and suppuration prevented.

In our present issue, we publish a communication from Surgeon Chisolm, of Charleston, South Carolina, advocating the extension of this principle to the treatment of all gun-shot wounds. The suggestion comes from one whose opinion deservedly commands the public attention, and there is about the proposed method so much plausibility, that we devote a little space to its consideration.

Many reliable observations during the progress of this war force us to conclude that gun-shot wounds do, under certain circumstances and when least to be expected, get well by the first intention.* This fact gives great weight to such a proposition as the one under consideration; for if the track of the ball is inclined to heal without suppuration, it is conceded that the process of paring away the bruised edges of the orifices, the bringing together of the fresh surfaces, and the firm bandaging of the whole limb, would greatly favor such good intention and expedite the cure.

The true difficulty, however, which arises here, and which must always be urged against such sweeping propositions in surgery or medicine is, how to tell when we may expect such a benign tendency on the part of the wounded tissues. Every observer knows that profuse suppuration occurs oftentimes when there is apparently but little laceration of the soft parts and no evidence of foreign matters interfering with the restorative forces, upon which Surgeon Chisolm and Doctor Howard so much rely. In one instance, we see a fracture of the femur in the upper-third healing by the first intention, with shortening of two inches. In another, after the orifices have closed spontaneously in a simple wound of the buttocks, the author has had repeated abscesses to form along the track of the ball, the case closing with erysipelas and pyemic fever.

With such contradictory results before him, the cautious surgeon should consider well the general condition of his patient, the character of the wound, the size of the ball and its probable velocity as it passed throcgh the soft parts, before expecting too much from the proposed treatment by hermetically sealing the orifices of the wound.

When the wound is made by a ball at its maximum of velocity passing cleanly through the soft parts, without carrying in any fragments of wad or clothing, or fracturing bone in its route; in such instances, Surgeon Chisolm's suggestion will be, in all probability, entirely successful, for it is founded upon this important truth—that the action of the oxygen of the air upon unprotected animal tissues is hurtful and favors the process of suppuration; but remembering the many instances of suppurative inflammation in sealed cavities, lumbar abscesses, cold abscesses, and the numerous imposthumes of our past experience, we should fear to adopt in all its force the following animated language of our esteemed correspondent:

"If these incisions are carefully brought together by sutures, and the limb or trunk be supported by a roll of bandage, they will rapidly unite by the first intention, converting the track, however long it may be, into a subcutaneous wound, which will heal rapidly, without suppuration, by a process known as the remodelling process, which is well exemplified in the subcutaneous division of tendons. * * * Should quick union be obtained, comparatively speaking, a cure is effected in from forty-eight to seventy-two hours, which, under the usual conditions, will require weeks, and may not be cured for months; the patient escaping all of the dangers from hospital gangrene, secondary hemorrhage, and the common one of protracted suppuration, followed often by contracted limbs; and, besides a large saving of human life, the government saving in hospital accommodation, in supplies for the wounded, in the avoidance of long and repeated furloughs, and in the speedy restoration of the wounded, to add to the effective strength of the army in the field. Nearly the entire treatment of the wounded should be perfected at the field infirmaries. In the few days following a battle, while the wounded are awaiting transportation, the outer orifices of their wounds can be healed, and the cases, now being beyond all danger, could be directly furloughed for a short period without going into hospital."

* Some of the most interesting of these cases are well thrown together in the paper of Surgeon Michel, compiled from the Transactions of Army and Navy Surgeons, see Vol. I., No. 8.

Surgeons in the field are respectfully requested to test the efficacy of the above method of rapidly healing gun-shot wounds, and report their results to the Surgeon-General's office.

---

### *Grand Summary of the Sick and Wounded of the Confederate States Army under Treatment during the Years* 1861 *and* 1862.

The immense mass of reports from the army medical corps for the first two years of the war, has been carefully winnowed and digested under the supervision of the Surgeon-General, and a general summary laid before Congress at its last session. Necessarily imperfect as these statistics are, they show at a glance the herculean labors performed by the medical staff. Gathered up as the army was from homes of peace to meet the throng of the invading enemy, the amount of sickness surpasses anything on record, while the ratio of mortality is far below the usual average. The medical department—without resources of any sort, without organization, without hospitals or the furniture to equip them, without transportation, self-depending and almost self-sustaining—assumed the enormous burden which is reached in the accompanying figures, containing a few of the leading diseases of the years 1861 and 1862, and faithfully conducted their task to a satisfactory conclusion. We should be prepared to make every allowance for the many imperfect statements and confused or irregular reports consequent on the confusion attendant on these faithful public officers, whilst fighting manfully and but half equipped with such a torrent of disease In our next issue, when the more complete records of the year 1863 will be presented to the reader, a comparison, not without interest to all, can then be instituted.

GENERAL RESUMÉ.

From all the reports now on file in the Surgeon-General's office for the years 1861 and 1862, exclusive of the few scattering ones which have reached us from the Trans-Mississippi Department, we are enabled to sum up the sickness and mortality occurring in our armies as follows:

*Continued Fevers.*—Field reports, 36,746 cases and 5,205 deaths. Hospital reports, 40,565 cases and 7,020 deaths.

*Paroxysmal Fevers.*—Field reports, 115,415 cases and 848 deaths. Hospital reports, 49,311 cases and 485 deaths.

*Eruptive Fevers*—Field, 44,438 cases and 1,036 deaths Hospitals, 32,755 cases and 1,238 deaths.

*Diarrhœa and Dysentery.*—Field, 226,828 cases and 1,696 deaths. Hospitals, 86,506 cases and 1,658 deaths.

*Pulmonary Affections.*—Field, 42,204 cases, 3,534 deaths, and 4,538 discharges from service. Hospitals, 36,988 cases, 4,538 deaths, and 1,135 discharges.

*Rheumatism.*—Field, 29,334 cases and 1,142 discharges. Hospitals, 30,438 cases and 700 discharges.

*Gun-Shot Wounds.*—Field, 29,569 cases, 1,623 deaths, and 493 discharges. Hospitals, 47,724 cases, 2,618 deaths, and 742 discharges. Killed in battle, 8,087.

*All other Diseases.*—Field, 324,321 cases and 2,278 deaths. Hospital, 123,402 cases and 1,802 deaths.

Whole number of cases exhibited in the field reports during 1861 and 1862 was 848,555; of which 16,220 died and 10,455 were discharged from service. There were admitted in hospitals for the same period 447,689 cases; of which 19,359 died and 6,485 were discharged.*

We learn also from these reports that of all the cases represented as originating in the field, but 108,068 were sent to general hospitals. If this be so, the large number received into hospitals, as shown by their own returns, can only be accounted for in the repeated transfer of patients during convalescence from one hospital to another.†

It is greatly to be regretted that the interest naturally felt in medico-vital statistics, when based on accurate and reliable data, can scarcely be claimed for what is offered in this paper. Still, if it have but the effect of directing the attention of medical officers more closely and carefully to the reports required of them, it will not be altogether without good results. This is but a beginning, and the next annual report, that for 1863, will doubtless embody many facts of a much more useful and interesting character.

---

*To the Reader.*

The publishers, at great cost of time and labor, have secured a supply of paper sufficient to justify an enlargement of the Journal, commencing with the present number. These additional expenses will render it necessary to increase the price to twenty dollars for the year's subscription, which will be charged to all subscribers from and after this date. The present list will be furnished with the Journal without additional cost until the close of the year.

With more space, the editor hopes to give to his readers an additional amount of information, enough to justify the increased cost, and, having successfully opened a regular communication with the European journals, this reasonable expectation should not be frustrated. He cordially desires the assistance of the Southern profession, without which, success cannot be achieved, and shall continue to labor, with an eye singly to advance the interests of medical science.

Hereafter, there will appear in each number a Monthly Bulletin from the Surgeon-General's office, in which all important changes in the department, new regulations, and general orders will be officially published. The medical officers of the army will here find another feature of interest to advance the value of the work.

* [What becomes of the loud outcry from the army against the general hospitals for discharging men in such large numbers? The figures show the proportion as 10 to 6 between field and hospital discharges. They also show still more conclusively that the number of discharged men is too small. The average number of recruits received in the British and French armies is generally about one-third of those who apply. Here, we find that in our army, collected *en masse* during the first two years of a most arduous war, only 16,000 men were discharged with a grand aggregate of nearly 1,300,000 cases.—ED.]

† [It should also be remembered that great numbers of sick and wounded have found their way into the general hospitals directly from the field, without passing through the hands of the regimental surgeons. This has especially occurred in the campaigns around Richmond, where the general hospitals have been only a few miles to the rear of the battle-fields.—ED.]

